# Performance of highly sensitive and conventional rapid diagnostic tests for clinical and subclinical *Plasmodium falciparum* infections, and *hrp2*/*3* deletion status in Burundi

**DOI:** 10.1371/journal.pgph.0000828

**Published:** 2022-07-28

**Authors:** David Niyukuri, Denis Sinzinkayo, Emma V. Troth, Colins O. Oduma, Mediatrice Barengayabo, Mireille Ndereyimana, Aurel Holzschuh, Claudia A. Vera-Arias, Yilekal Gebre, Kingsley Badu, Joseph Nyandwi, Dismas Baza, Elizabeth Juma, Cristian Koepfli

**Affiliations:** 1 Doctoral School, University of Burundi, Bujumbura, Burundi; 2 South African DSI-NRF Centre of Excellence in Epidemiological Modelling and Analysis, Stellenbosch University, Stellenbosch, South Africa; 3 National Malaria Control Program, Bujumbura, Burundi; 4 Eck Institute for Global Health and Department of Biological Sciences, University of Notre Dame, Notre Dame, Indiana, United States of America; 5 Kenya Medical Research Institute, Kisumu, Kenya; 6 Kwame Nkrumah University of Science and Technology, Kumasi, Ghana; 7 National Institute of Public Health, Bujumbura, Burundi; 8 WHO Country Office, Bujumbura, Burundi; 9 WHO African Region, Accra, Ghana; Menzies School of Health Research, AUSTRALIA

## Abstract

Rapid diagnostic tests (RDTs) are a key tool for the diagnosis of malaria infections among clinical and subclinical individuals. Low-density infections, and deletions of the *P*. *falciparum hrp2*/*3* genes (encoding the HRP2 and HRP3 proteins detected by many RDTs) present challenges for RDT-based diagnosis. The novel Rapigen Biocredit three-band *Plasmodium falciparum* HRP2/LDH RDT was evaluated among 444 clinical and 468 subclinical individuals in a high transmission setting in Burundi. Results were compared to the AccessBio CareStart HRP2 RDT, and qPCR with a sensitivity of <0.3 parasites/μL blood. Sensitivity compared to qPCR among clinical patients for the Biocredit RDT was 79.9% (250/313, either of HRP2/LDH positive), compared to 73.2% (229/313) for CareStart (*P* = 0.048). Specificity of the Biocredit was 82.4% compared to 96.2% for CareStart. Among subclinical infections, sensitivity was 72.3% (162/224) compared to 58.5% (131/224) for CareStart (*P* = 0.003), and reached 88.3% (53/60) in children <15 years. Specificity was 84.4% for the Biocredit and 93.4% for the CareStart RDT. No (0/362) *hrp2* and 2/366 *hrp3* deletions were observed. In conclusion, the novel RDT showed improved sensitivity for the diagnosis of *P*. *falciparum*.

## Introduction

Accurate diagnosis and treatment of malaria infections is a key pillar for control. Over the last decade, rapid diagnostic tests (RDTs) have become widely used for diagnosis. RDTs are lateral flow devices that detect parasite-specific proteins by immunohistochemistry. No laboratory infrastructure is required for RDT use, they can be used with minimal training, and results are available within 10–20 minutes [1, 2]. The sensitivity of RDTs for the diagnosis of clinical cases is similar or better than field microscopy, the only other method for diagnosis in health centers [3, 4]. RDTs are used in many peripheral health centers where microscopy is not available, and by community health workers. In 2020, nearly 300 million RDTs were used by malaria control programs [5].

In addition to clinical infections diagnosed at health centers, subclinical infections present a major challenge for control. While not causing febrile illness, subclinical infections can result in more subtle health impacts, e.g. anemia [6]. Using different approaches, several studies found that over 50% of transmission originates from asymptomatic infections [7–9]. A study conducted in western Kenya estimated 95% of transmission stemming from subclinical carriers [10]. RDTs are the key diagnostic tool for strategies to target the subclinical reservoir, for example through active [11, 12] or reactive case detection [13, 14],

Among clinical and subclinical infections, a proportion of infections are below the limit of detection of RDTs and remain untreated. These untreated infections might result in extended periods of illness, and sustain onward transmission. More sensitive RDTs might be needed to achieve further gains in malaria control and elimination.

Apart from low parasite density, deletions of the *hrp2* and *hrp3* genes are a threat to diagnosis by RDT. The most sensitive RDTs for *P*. *falciparum* detect the HRP2 and HRP3 proteins. Deletions of these genes have been initially described in Peru [15] and later in multiple countries, and result in false-negative RDTs even when parasite density is high. In Africa, deletions are particularly prevalent around the Horn of Africa [16–18], and have been described in multiple other countries, e.g. the Democratic Republic of Congo [19], and Ghana [20]. Alternative diagnostic targets include Plasmodium Lactate Dehydrogenase (pLDH) or Aldolase. These proteins are essential and thus genes cannot be deleted, but sensitivity of diagnosis is lower compared to HRP2 [21]. The WHO recommends regular surveillance of *hrp2*/*hrp3* deletion to select the optimal tool for diagnosis [22].

The level of malaria transmission in Burundi is among the highest in the world [23, 24]. In contrast to the trend in many other countries in sub-Saharan Africa, the number of cases has increased in the last two decades [24]. The increase was observed despite an increase in the number of health centers offering diagnosis and treatment, and increase in testing, and roll-out of interventions such as bed nets and indoor residual spraying (IRS) [24]. To understand the benefit of a novel, highly sensitive RDT, suspected clinical cases presenting to a health center, and subclinical community members were tested by the novel RDT, an established RDT, and results were compared to highly sensitive qPCR. *P*. *falciparum* positive infections were typed for *hrp2* and *hrp3* deletions.

## Study population and methods

### Ethical approval

Informed written consent was collected from each individual, or, in the case of minors, from the legal guardian prior to sample collection. This study was approved by the Comité National d’Ethique pour la protection des êtres humains sujets de la recherche biomédicale et comportementale of Burundi (approval no. CNE/03/2021), and the University of Notre Dame Institutional Review Board (approval no. 21-02-6446).

### Rapid diagnostic tests

The Biocredit Malaria Ag Pf (pLDH/HRPII) RDT (lot no. H052A001DA), manufactured by Rapigen, contains two test bands, one for HRP2 and one for LDH, in addition to the control band. Plasmodium Lactate Dehydrogenase (pLDH) is an enzyme of the glycolytic pathway and essential for the parasite. Histidine Rich Protein 2 and 3 are highly expressed proteins. Their function is not well understood [2]. Deletions of the *hrp2* and *hrp3* genes result in false-negative RDTs. The test was evaluated previously among microscopy-positive individuals and showed very high agreement with diagnosis by microscopy [25]. It has not been not evaluated among clinical patients that might harbor submicroscopic infections, or subclinical individuals. Given the separated HRP2 and LDH bands, the test can be used for surveillance of possible *hrp2* deletion. Differences in sensitivity of HRP2 and LDH can be studied using a single test. This test meets critical WHO criteria and is currently undergoing WHO prequalification [26].

As comparison, the AccessBio CareStart Malaria Pf (HRP2) RDT was used (lot no. MO19H71). This HRP2-based RDT is WHO pre-qualified [26] and has been in use for approximately ten years [27–29]. It is the main RDT used by health systems in multiple African countries [20, 30].

Testing procedures are identical for both RDTs and are explained in detail e.g. in [31]. A droplet of blood (approximately 5 μL), collected either by finger prick or phlebotomy, is put onto a specific spot on the test kit, followed by 2–3 drops of assay diluent buffer on a separate spot. Results are read after 15–20 minutes. The cost for either test is approximately 1 USD.

### Sample collection and RDT diagnosis

Burundi experiences high, year-round transmission of *P*. *falciparum* malaria with moderate seasonality. Samples were collected in Cibitoke Province in northwestern Burundi. Cibitoke Province borders Rwanda and the Democratic Republic of Congo (DRC). Samples were collected during the dry, lower transmission season in June 2021. Clinical samples were collected in health centers. All patients presenting for malaria diagnosis were invited to join the study. Individuals found positive were treated according to national guidelines. Subclinical samples were collected on a market, with all individuals present invited to join the study.

Approximately 200 μL blood was collected by finger prick into EDTA tubes. Two RDTs were run on site. Remaining blood was placed into -20°C storage every evening and kept at -20°C until DNA extraction.

### *P*. *falciparum* qPCR and *hrp2/3* deletion typing

DNA was extracted using the Macherey-Nagel NucleoMag Blood 200 μL kit according to manufacturer’s instructions. This kit yields very high DNA recovery, resulting in a low limit of detection [32]. DNA was extracted from 200 μL blood and eluted in 100 μL elution buffer. qPCR was done in a total volume of 12 μL, including 2 μL DNA, corresponding to 4 μL blood. The *P*. *falciparum varATS* assay was used. This assay targets a multicopy gene that is present in approximately 60 copies per parasite, of which approximately 20 copies are amplified with the primers and probe used. It thus offers very high sensitivity of <0.3 parasites/μL blood [33]. qPCR conditions are given in S1 File. For absolute quantification of parasite density, a standard curve derived from DNA from cultured 3D7 parasites and quantified by droplet digital PCR (ddPCR) was run along samples. Samples positive for *P*. *falciparum* at a density of approximately >5 parasites/μL were typed for *hrp2* and *hrp3* deletion by ddPCR [34]. In this assay, *hrp2* or *hrp3* and a control gene (*serine-tRNA ligase*) are quantified in a single tube with very high specificity, thus providing highly accurate data on deletion status [34]. The assay amplifies parts of *hrp2* exon 2 that encodes for the antigen detected by the RDT. The amplified region is deleted in all reported cases where *hrp2* deletion breakpoints were studied [35]. Assay conditions are given in S1 File.

### Data analysis

The sample size was determined to be able to detect a 5% difference in sensitivity with 95% confidence. In the absence of any preliminary data, a prevalence of infection of 50% was assumed, yielding the largest sample size of n = 377. The number of individuals sampled (n = 444 clinical and n = 468 subclinical) was above the minimum target, these numbers were reached out of convenience.

The following performance characteristics were determined for both RDTs, and the HRP2 and LDH bands on the Biocredit RDT separately as well as in combination: (i) sensitivity: the number of infections detected by an RDT divided by the total number of infections detected by qPCR as gold standard. (ii) Specificity: the proportion of negative RDTs among individuals that tested negative by qPCR. (iii) Limit of detection (LoD): LoD was defined as the lowest parasite density where a qPCR-positive infection would be detected with 95% probability. To determine the limit of detection (LoD) of each RDT target, logistic regression analysis was conducted. (iv) Area under the receiver operating characteristic curve (ROC) curve (AUC): AUC was calculated with a nonparametric analysis using 1000 bootstrap replications, with log10 transformed parasite density by qPCR as classification variable. Diagnostic accuracy was considered excellent if AUC was >0.9, and very good for AUC values of >0.8 to <0.9 [36].

As parasite density distributions were skewed, geometric mean densities are given whenever densities are reported. CI95 stands for 95% confidence interval. All data can be found in S1 Data.

## Results

### Study population demographics

Demographic data of study participants are given in Table 1. Due to the convenience sampling strategy, the subclinical study population does not represent the general population. Only 27/468 subclinical individuals were children below 5 years of age, and only 73/468 were children aged 5–15 years. The majority of those sampled were female.

**Table 1:** Demographics of study population and prevalence and test positivity (by qPCR and RDT) by age group and gender.

Clinical		N	Test positivity by qPCR	*P*	Test positivity by RDT	*P*	RDT sensitivity	*P*
Age group	0–5	158 (35.6%)	62.0%		49.4%		72.4%	
	5–15	99 (22.3%)	82.8%	0.002	86.9%	<0.001	96.3%	<0.001
	>15	187 (42.1%)	71.1%		58.3%		75.2%	
Gender	Male	181 (59.2%)	67.4%		62.7%		82.0%	
	Female	263 (40.8%)	72.6%	0.236	59.7%	0.514	78.5%	0.460
**Subclinical**		N	Prevalence by qPCR	*P*	Prevalence by RDT	*P*	RDT sensitivity	*P*
Age group	0–5	27 (5.8%)	51.8%		55.6%		78.6%	
	5–15	73 (15.6%)	63.0%	0.015	67.1%	<0.001	91.3%	0.003
	>15	368 (78.6%)	44.7%		36.8%		66.5%	
Gender	Male	217 (46.5%)	50.7%		39.6%		75.5%	
	Female	250 (53.5%)	45.6%	0.272	46.1%	0.158	69.3%	0.303

For RDT data, results from the Biocredit test were included, with either band (HRP2 or LDH) positive counting as a positive test. Test positivity and prevalence by RDT includes individuals that were RDT positive but qPCR negative, resulting in higher test positivity or prevalence by RDT than qPCR in some groups. RDT sensitivity is calculated as proportion of RDT positive individuals among those positive by qPCR. *P*-values to compare groups were calculated by Chi-square test.

### RDT sensitivity and specificity

**Fig 1:**
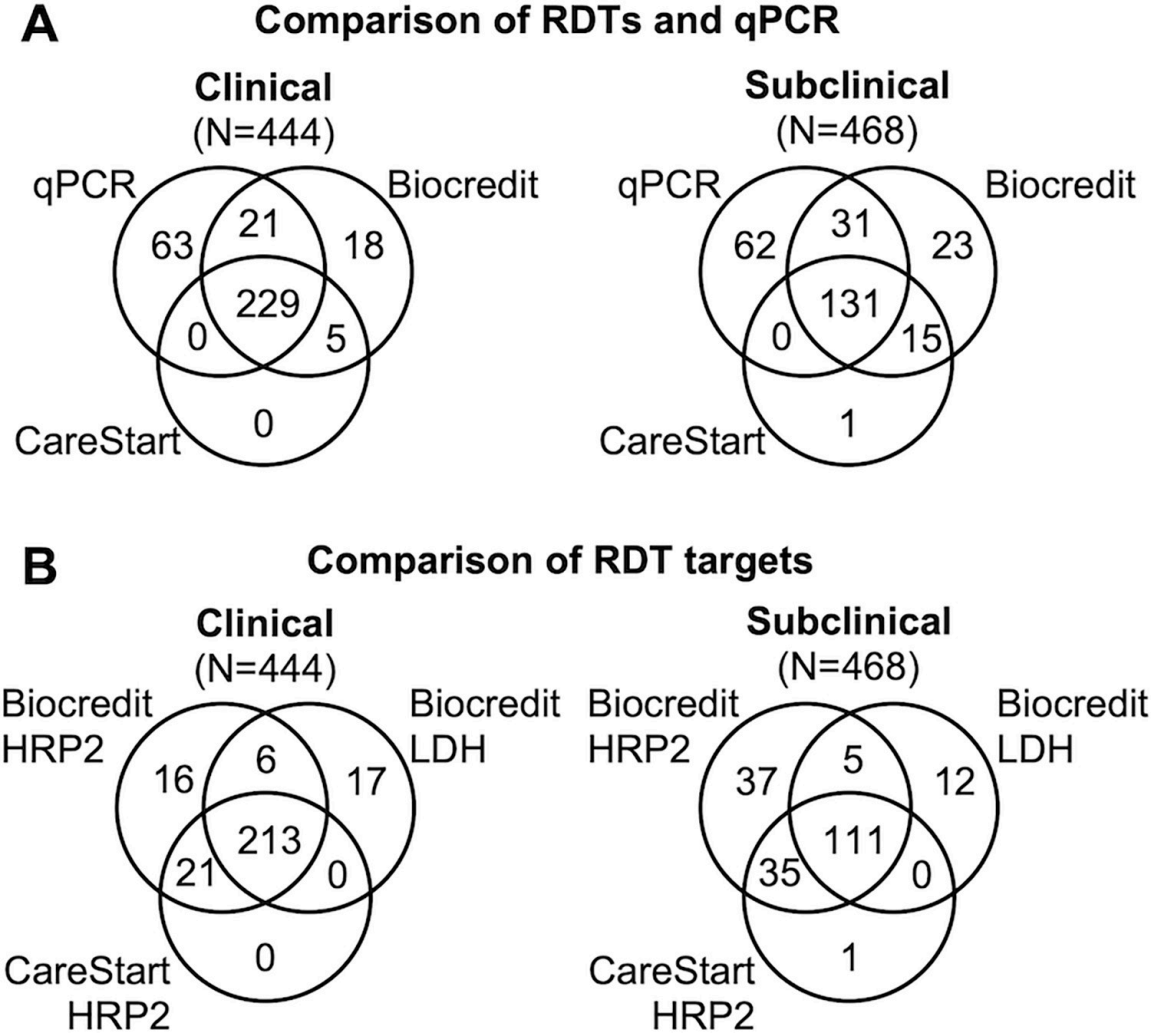
Correspondence among qPCR, and different RDTs and RDT targets. For panel A, when either band on the Biocredit RDT was positive it was recorded as positive. Panel B includes false-positive RDTs that were negative by qPCR.

444 clinical patients presenting to a health center were enrolled. By qPCR, *P*. *falciparum* positivity was 70.5% (313/444). Mean parasite density by qPCR was 727.4 parasites/μL (CI95: 405.9, 1303.6). Counting either band (HRP2 or LDH) positive, the sensitivity of the Biocredit RDT was 79.9% compared to qPCR (250/313 of infections detected, Fig 1A, Tables 2 and 3). 23 individuals tested positive by RDT, but negative by qPCR, resulting in a specificity of 82.4%.

**Table 2:** Limit of detection, AUC, and sensitivity and specificity of RDTs as compared to qPCR.

		Clinical			Subclinical		
	LoD [parasites/μL] [CI95]	Sensitivity [CI95]	Specificity [CI95]	AUC [CI95]	Sensitivity [CI95]	Specificity [CI95]	AUC [CI95]
Biocredit any band	34 [17, 82]	79.9% [75.0, 84.0]	82.4% [74.9, 88.1]	0.939 [0.913, 0.964]	72.3% [66.1, 77.8]	84.4% [79.3, 88.5]	0.862 [0.811, 0.913]
Biocredit HRP2	42 [22, 96]	77.6% [72.7, 81.9]	90.1% [83.6, 94.2]	0.955 [0.935, 0.975]	71.0% [64.7, 76.6]	88.1% [83.4, 91.6]	0.863 [0.813, 0.914]
Biocredit LDH	290 [159, 617]	71.9% [66.6, 76.6]	91.6%[85.4, 95.3]	0.965 [0.945, 0.984]	50.0% [43.5, 56.5]	93.4% [89.6, 96.0]	0.907 [0.869, 0.944]
CareStart HRP2	178 [94, 399]	73.2% [68.0, 77.8]	96.2% [91.1, 98.4]	0.962 [0.943, 0.981]	58.5% [51.9, 64.8]	93.4% [89.5, 96.0]	0.867 [0.818, 0.916]

**Table 3:** Number of positives by RDT vs. qPCR, and by Biocredit vs. CareStart.

A) Clinical infections				B) Subclinical infections			
		Biocredit (any band)				Biocredit (any band)	
		Negative	Positive			Negative	Positive
qPCR	Negative	108	23	qPCR	Negative	206	38
	Positive	63	250		Positive	62	162
		Carestart				Carestart	
		Negative	Positive			Negative	Positive
qPCR	Negative	126	5	qPCR	Negative	228	16
	Positive	84	229		Positive	93	131
		Biocredit (any band)				Biocredit (any band)	
		Negative	Positive			Negative	Positive
Carestart	Negative	171	39	Carestart	Negative	267	54
	Positive	0	234		Positive	1	146

Among 468 subclinical individuals enrolled, 47.9% (224/468) were positive by qPCR. Mean parasite density was 22.9 parasites/μL (CI95: 13.1, 40.3). The sensitivity of the Biocredit test was 72.3% compared to qPCR (162/224, Fig 1A, Tables 2 and 3). 38 individuals were positive by RDT but negative by qPCR, resulting in a specificity of 84.4% (194/230).

The novel Biocredit RDT was compared against the CareStart RDT, an established product routinely used in multiple countries (Fig 1A, Table 3). The CareStart RDT showed a sensitivity of 73.2% (229/313) compared to qPCR among clinical individuals and thus was slightly less sensitive than the Biocredit RDT with a sensitivity of 79.9% (*P*<0.001, Table 2). Five individuals tested positive by CareStartRDT but negative by qPCR, resulting in a specificity of 96.2%. Among subclinical individuals, sensitivity of the CareStart was 58.5% (131/224), and thus substantially lower than the Biocredit at 72.3%. Specificity was 93.4% (228/244, *P*<0.001, Table 2).

On the Biocredit RDT, the HRP2 band was more sensitive than the LDH band (Fig 1B). Among clinical individuals, sensitivity of HRP2 was 77.6% (243/313), compared to 71.9% (225/313) for the LDH band (*P*<0.001, Table 2). Specificity was 90.1% (118/131) for HRP2, and 91.6% (120/131) for LDH (Table 2). Among subclinical individuals, the difference in sensitivity between HRP2 and LDH was more pronounced than among clinical infections. Sensitivity of HRP2 was 71.0% (159/224), compared to 50.0% (112/224) for LDH (*P*<0.001, Table 2). Specificity for HRP2 only was 88.1% (215/244), and for LDH only it was 93.4% (214/230, Table 2). Low correspondence between false-positive HRP2 and LDH bands was observed. Among 61 false-positive RDTs (clinical and subclinical combined), only 8 were false-positive for both targets, 34 were positive for HRP2 only, and 19 for LDH only.

**Fig 2:**
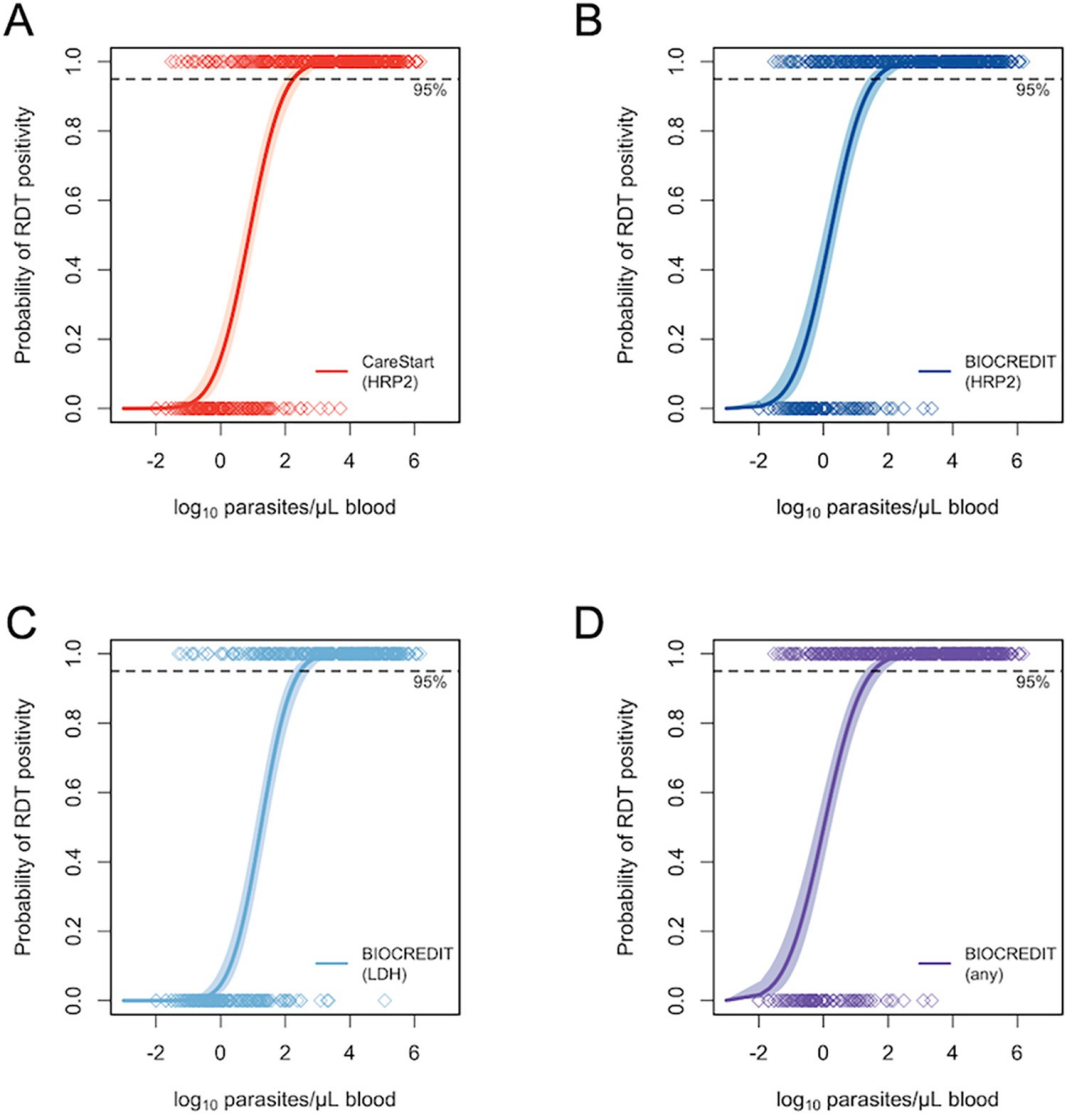
Limit of Detection (LoD) of each RDT and RDT target in relation to parasite density. Parasite density (by qPCR) of RDT positive and RDT negative samples is shown along the top and bottom X-axis. Shaded lines show 95% confidence intervals. A) Biocredit (any band) B) Biocredit HRP2 C) Biocredit LDH D) CareStart HRP2.

The LoD, defined as the lowest parasite density that could be detected with 95% probability, was 34 parasites/μL (CI95 17, 82) for the Biocredit RDT (any band), 42 parasites/μL (CI95 22, 96) for the Biocredit HRP2 band, 290parasites/μL (CI95 159, 617) for the Biocredit LDH band, and 178 parasites/μL (CI95 94, 399) for CareStart (Table 2, Fig 2).

The diagnostic accuracy as measured by AUC analysis was excellent (>0.9) among clinical samples for both RDTs and each target on the Biocredit RDT (Table 2). Among subclinical individuals, diagnostic accuracy was very high (>0.8) for all RDTs, and reached 0.907 for the LDH band on the Biocredit.

### *hrp2*/*3* deletion typing

362 *P*. *falciparum* positive samples were successfully typed for *hrp2* deletion, and 366 were successfully typed for *hrp3* deletion. No *hrp2* deletions were observed. Two samples carried *hrp3* deletion, one each collected from a clinical and subclinical patient. The subclinical individual was negative by all RDTs at a density of 39 parasites/μL by ddPCR. The clinical individual was RDT positive at a density of 136,000 parasites/μL.

### Demographic risk factors for infection

Prevalence of subclinical infection, and test positivity rate peaked in children aged 5 to 15 years, both by qPCR and RDT (Table 1). Parasite densities decreased with age in clinical and subclinical infections (clinical: 9.7% decrease per year, *P*<0.001; subclinical: 4.6% decrease per year, *P* = 0.003). As a result, RDT sensitivity tended to be higher in children than adults. Among clinical infections, sensitivity of the Biocredit RDT (any band) was 83.3% (150/180) in children below 15 years compared to 75.2% (100/133) in adults aged 15 and older (*P* = 0.076). Among subclinical infections, sensitivity was 88.3% (53/60) in children and 66.5% (109/164) in adults (*P* = 0.001).

No significant differences were observed between men and women in prevalence by qPCR or RDT, or in RDT sensitivity (Table 1).

## Discussion

This study found high sensitivity of a novel RDT for the diagnosis of *P*. *falciparum* infections in clinical and subclinical individuals in highly endemic setting in Burundi. Using a highly sensitive qPCR with a limit of detection of <0.3 parasites/μL blood as gold standard, the RDT detected 80% of clinical and 72% of subclinical infections. The novel Biocredit RDT performed substantially better compared to the established CareStart RDT that is used by malaria control programs in multiple countries.

Due to the acquisition of natural immunity after repeated infections, parasite densities in children are highest, they are at highest risk of severe malaria, and in high transmission settings they are an important reservoir of transmission. The convenience sampling on a market resulted in an underrepresentation of children among subclinical individuals, with only 21% (100/468) study participants being below 15 years of age, compared to approximately 45% in the general population [37]. In the small group of 60 children that were positive by qPCR, the Biocredit RDT detected 88% of infections. Testing a group with an age distribution more representative of the general population, it is expected that the overall sensitivity of the RDT would be even higher than the 72% measured in the present study.

Compared to the established CareStart RDT, the LoD (defined as the parasite density where the probability of a positive RDT is 95%) of the Biocredit RDT was 5-fold lower. The LoD of the Biocredit HRP2-band only was 4-fold lower compared to the CareStart RDT, and the LoD of the Biocredit LDH-band only was moderately higher than the one of the CareStart. Of note, differences in the protocols used for DNA extraction [32], and in the external standards used for qPCR [38], result in pronounced differences in parasite quantification. Results from different studies are thus not directly comparable. We extracted DNA from whole blood and used an extraction protocol that yielded fourfold more DNA than another common protocol. Further, extraction from whole blood yielded tenfold more DNA than from DBS [32]. Using these protocols resulted in high DNA recovery, and thus in higher LoDs.

No *hrp2* deletions were detected in the study population. Differences in the number of tests positive by LDH and HRP2 are thus fully caused by differences in the LoD of these targets. On the Biocredit RDT, HRP2 was substantially more sensitive than LDH. Where no deletions are present, HRP2-based RDTs remain the most sensitive diagnostic target for RDTs. Among clinical infections, sensitivity of the Biocredit LDH band was very similar to the AccessBio HRP2-based RDT (72% vs. 73%). Countries currently using the Carestart HRP2 RDT that switch to non-HRP2-based RDTs due to high levels of deletion can expect similar sensitivity of diagnosis in clinical settings using the new test as with previous HRP2-based RDTs.

A high rate of false-positive RDTs was observed, and consequently a relatively low specificity. Antigens can persist after parasite clearance [39], though LDH levels were shown to follow parasite density closely [39]. Antigens persisting after treatment were shown to be a major cause of false-positive RDTs [40]. Recent treatment was not assessed in this study, thus this factor could not be assessed as a cause for false-positive RDTs. False-positive RDTs can also be the result of non-*Plasmodium* infections [41, 42] and non-infectious diseases [43]. Most false-positive Biocredit RDTs were positive by one target only, either HRP2 or LDH. Using an RDT with two bands reduces specificity compared to a single-target test.

Half of the mostly adult study population in the study site in Burundi’s north-west carried subclinical *P*. *falciparum* infections. These individuals are expected to be an important source of transmission [7–10]. Interventions targeting the asymptomatic reservoir might be needed to reduce transmission. The high sensitivity of the Biocredit RDT to diagnose subclinical infections opens up new avenues for malaria control, e.g. through mass screen and treat campaigns.

In conclusion, the Biocredit RDT offers high sensitivity for the diagnosis of clinical and subclinical infections. Parasite density distributions differ across transmission intensities, and more subpatent infections are generally observed as transmission declines [44, 45]. Studies in regions of medium and low transmission will be required to determine sensitivity and specificity in these populations. Given the absence of *hrp2* deletions, HRP2-based diagnostic remains appropriate in the site of the current study. Surveillance for *hrp2* and *hrp3* deletions will be required in other sites across Burundi.

## Supporting information

S1 File Laboratory protocols: *P*. *falciparum var*ATS qPCR, *hrp2* deletion, *hrp3* deletion. (DOCX)

S1 Data Database. (TXT)

## References

[1] World Health Organisation. How malaria RDTs work. Available from: https://www.who.int/teams/global-malaria-programme/case-management/diagnosis/rapid-diagnostic-tests/how-malaria-rdts-work.

[2] Poti KE, Sullivan DJ, Dondorp AM, Woodrow CJ. HRP2: Transforming Malaria Diagnosis, but with Caveats. Trends Parasitol. 2020;36(2):112–26. Epub 2019/12/19. doi: 10.1016/j.pt.2019.12.004 . 31848119

[3] Afriyie SO, Addison TK, Gebre Y, Mutala A-H, Antwi KB, Abbas DA, et al. Accuracy of diagnosis among clinical malaria patients: comparing microscopy, RDT, and a highly sensitive quantitative PCR and the implication of submicroscopic infections. 2022. doi: 10.21203/rs.3.rs-1510630/v1 PMC9985253 36870966

[4] Pasaribu AP, Nasution IS, Sembiring K, Fahmi F, Pasaribu S. Comparison of the performance of the CareStart Malaria Pf/Pan Combo test and field microscopy in the diagnosis of Plasmodium vivax malaria in North Sumatera, Indonesia. Malar J. 2022;21(1):27. Epub 2022/01/31. doi: 10.1186/s12936-022-04057-1 ; PubMed Central PMCID: PMC8800299. 35093067 PMC8800299

[5] World Health Organisation. World malaria report 2021. Geneva: 2021.

[6] de Mast Q, Brouwers J, Syafruddin D, Bousema T, Baidjoe AY, de Groot PG, et al. Is asymptomatic malaria really asymptomatic? Hematological, vascular and inflammatory effects of asymptomatic malaria parasitemia. J Infection. 2015;71(5):587–96. doi: 10.1016/j.jinf.2015.08.005 WOS:000368479700011. 26304688

[7] Goncalves BP, Kapulu MC, Sawa P, Guelbeogo WM, Tiono AB, Grignard L, et al. Examining the human infectious reservoir for Plasmodium falciparum malaria in areas of differing transmission intensity. Nat Commun. 2017;8(1):1133. Epub 2017/10/28. doi: 10.1038/s41467-017-01270-4 ; PubMed Central PMCID: PMC5658399. 29074880 PMC5658399

[8] Ouedraogo AL, Goncalves BP, Gneme A, Wenger EA, Guelbeogo MW, Ouedraogo A, et al. Dynamics of the Human Infectious Reservoir for Malaria Determined by Mosquito Feeding Assays and Ultrasensitive Malaria Diagnosis in Burkina Faso. J Infect Dis. 2016;213(1):90–9. Epub 2015/07/05. doi: 10.1093/infdis/jiv370 . 26142435

[9] Tadesse FG, Slater HC, Chali W, Teelen K, Lanke K, Belachew M, et al. The Relative Contribution of Symptomatic and Asymptomatic Plasmodium vivax and Plasmodium falciparum Infections to the Infectious Reservoir in a Low-Endemic Setting in Ethiopia. Clin Infect Dis. 2018;66(12):1883–91. Epub 2018/01/06. doi: 10.1093/cid/cix1123 . 29304258

[10] Sumner KM, Freedman E, Abel L, Obala A, Pence BW, Wesolowski A, et al. Genotyping cognate Plasmodium falciparum in humans and mosquitoes to estimate onward transmission of asymptomatic infections. Nat Commun. 2021;12(1):909. Epub 2021/02/12. doi: 10.1038/s41467-021-21269-2 ; PubMed Central PMCID: PMC7875998. 33568678 PMC7875998

[11] Scott CA, Yeshiwondim AK, Serda B, Guinovart C, Tesfay BH, Agmas A, et al. Mass testing and treatment for malaria in low transmission areas in Amhara Region, Ethiopia. Malar J. 2016;15:305. Epub 2016/06/04. doi: 10.1186/s12936-016-1333-3 ; PubMed Central PMCID: PMC4890322. 27255330 PMC4890322

[12] Samuels AM, Odero NA, Odongo W, Otieno K, Were V, Shi YP, et al. Impact of Community-Based Mass Testing and Treatment on Malaria Infection Prevalence in a High-Transmission Area of Western Kenya: A Cluster Randomized Controlled Trial. Clin Infect Dis. 2021;72(11):1927–35. Epub 2020/04/24. doi: 10.1093/cid/ciaa471 . 32324850 PMC9059430

[13] Stuck L, Fakih BS, Al-Mafazy AH, Hofmann NE, Holzschuh A, Grossenbacher B, et al. Malaria infection prevalence and sensitivity of reactive case detection in Zanzibar. Int J Infect Dis. 2020;97:337–46. Epub 2020/06/14. doi: 10.1016/j.ijid.2020.06.017 ; PubMed Central PMCID: PMC8450816. 32534138 PMC8450816

[14] Searle KM, Katowa B, Musonda M, Pringle JC, Hamapumbu H, Matoba J, et al. Sustained Malaria Transmission despite Reactive Screen-and-Treat in a Low-Transmission Area of Southern Zambia. Am J Trop Med Hyg. 2020;104(2):671–9. Epub 2020/11/26. doi: 10.4269/ajtmh.20-0947 ; PubMed Central PMCID: PMC7866307. 33236715 PMC7866307

[15] Gamboa D, Ho MF, Bendezu J, Torres K, Chiodini PL, Barnwell JW, et al. A large proportion of P. falciparum isolates in the Amazon region of Peru lack pfhrp2 and pfhrp3: implications for malaria rapid diagnostic tests. PLoS One. 2010;5(1):e8091. Epub 2010/01/30. doi: 10.1371/journal.pone.0008091 ; PubMed Central PMCID: PMC2810332. 20111602 PMC2810332

[16] Berhane A, Anderson K, Mihreteab S, Gresty K, Rogier E, Mohamed S, et al. Major Threat to Malaria Control Programs by Plasmodium falciparum Lacking Histidine-Rich Protein 2, Eritrea. Emerg Infect Dis. 2018;24(3):462–70. Epub 2018/02/21. doi: 10.3201/eid2403.171723 ; PubMed Central PMCID: PMC5823352. 29460730 PMC5823352

[17] Golassa L, Messele A, Amambua-Ngwa A, Swedberg G. High prevalence and extended deletions in Plasmodium falciparum hrp2/3 genomic loci in Ethiopia. PLoS One. 2020;15(11):e0241807. Epub 2020/11/06. doi: 10.1371/journal.pone.0241807 ; PubMed Central PMCID: PMC7644029 adherence to PLOS ONE policies on data or materials sharing. 33152025 PMC7644029

[18] Iriart X, Menard S, Chauvin P, Mohamed HS, Charpentier E, Mohamed MA, et al. Misdiagnosis of imported falciparum malaria from African areas due to an increased prevalence of pfhrp2/pfhrp3 gene deletion: the Djibouti case. Emerg Microbes Infect. 2020;9(1):1984–7. Epub 2020/09/02. doi: 10.1080/22221751.2020.1815590 ; PubMed Central PMCID: PMC7534257. 32869688 PMC7534257

[19] Parr JB, Verity R, Doctor SM, Janko M, Carey-Ewend K, Turman BJ, et al. Pfhrp2-Deleted Plasmodium falciparum Parasites in the Democratic Republic of the Congo: A National Cross-sectional Survey. J Infect Dis. 2016. doi: 10.1093/infdis/jiw538 . 28177502 PMC6279174

[20] Amoah LE, Abankwa J, Oppong A. Plasmodium falciparum histidine rich protein-2 diversity and the implications for PfHRP 2: based malaria rapid diagnostic tests in Ghana. Malar J. 2016;15:101. Epub 2016/02/20. doi: 10.1186/s12936-016-1159-z ; PubMed Central PMCID: PMC4759916. 26891848 PMC4759916

[21] Li B, Sun Z, Li X, Li X, Wang H, Chen W, et al. Performance of pfHRP2 versus pLDH antigen rapid diagnostic tests for the detection of Plasmodium falciparum: a systematic review and meta-analysis. Arch Med Sci. 2017;13(3):541–9. Epub 2017/05/17. doi: 10.5114/aoms.2017.67279 ; PubMed Central PMCID: PMC5420633. 28507567 PMC5420633

[22] World Health Organisation. Response plan to *pfhrp2* gene deletions. Geneva: 2019.

[23] Lok P, Dijk S. Malaria outbreak in Burundi reaches epidemic levels with 5.7 million infected this year. BMJ. 2019;366:l5104. Epub 2019/08/11. doi: 10.1136/bmj.l5104 . 31399412

[24] Sinzinkayo D, Baza D, Gnanguenon V, Koepfli C. The lead-up to epidemic transmission: malaria trends and control interventions in Burundi 2000 to 2019. Malar J. 2021;20(1):298. Epub 2021/07/04. doi: 10.1186/s12936-021-03830-y ; PubMed Central PMCID: PMC8249825. 34215270 PMC8249825

[25] Park SH, Jegal S, Ahn SK, Jung H, Lee J, Na BK, et al. Diagnostic Performance of Three Rapid Diagnostic Test Kits for Malaria Parasite Plasmodium falciparum. Korean J Parasitol. 2020;58(2):147–52. Epub 2020/05/19. doi: 10.3347/kjp.2020.58.2.147 ; PubMed Central PMCID: PMC7231823. 32418383 PMC7231823

[26] World Health Organisation. Selecting and procuring malaria RDTs 2022 [cited 2022 April 12]. Available from: https://www.who.int/teams/global-malaria-programme/case-management/diagnosis/rapid-diagnostic-tests/selection-and-procurement.

[27] Maltha J, Gillet P, Bottieau E, Cnops L, van Esbroeck M, Jacobs J. Evaluation of a rapid diagnostic test (CareStart Malaria HRP-2/pLDH (Pf/pan) Combo Test) for the diagnosis of malaria in a reference setting. Malar J. 2010;9:171. Epub 2010/06/23. doi: 10.1186/1475-2875-9-171 ; PubMed Central PMCID: PMC2906498. 20565816 PMC2906498

[28] Moges B, Amare B, Belyhun Y, Tekeste Z, Gizachew M, Workineh M, et al. Comparison of CareStart HRP2/pLDH COMBO rapid malaria test with light microscopy in north-west Ethiopia. Malar J. 2012;11:234. Epub 2012/07/24. doi: 10.1186/1475-2875-11-234 ; PubMed Central PMCID: PMC3411439. 22818643 PMC3411439

[29] World Health Organisation. Malaria rapid diagnostic test performance: results of WHO product testing of malaria RDTs: round 8 (2016–2018). Geneva: 2018 978-92-4-151496-5.

[30] Teh RN, Sumbele IUN, Asoba Nkeudem G, Meduke DN, Ojong ST, Kimbi HK. Concurrence of CareStart Malaria HRP2 RDT with microscopy in population screening for Plasmodium falciparum infection in the Mount Cameroon area: predictors for RDT positivity. Trop Med Health. 2019;47:17. Epub 2019/03/15. doi: 10.1186/s41182-019-0145-x ; PubMed Central PMCID: PMC6397448. 30867636 PMC6397448

[31] World Health Organisation, The USAID Quality Assurance Project (QAP). How to use a rapid diagnostic test (RDT): A guide for training at a village and clinic level. 2010.

[32] Holzschuh A, Koepfli C. Tenfold difference in DNA recovery rate: systematic comparison of whole blood vs. dried blood spot sample collection for malaria molecular surveillance. Malar J. 2022;21(1):88. Epub 2022/03/17. doi: 10.1186/s12936-022-04122-9 . 35292038 PMC8922754

[33] Hofmann N, Mwingira F, Shekalaghe S, Robinson LJ, Mueller I, Felger I. Ultra-sensitive detection of Plasmodium falciparum by amplification of multi-copy subtelomeric targets. PLoS Med. 2015;12(3):e1001788. Epub 2015/03/04. doi: 10.1371/journal.pmed.1001788 ; PubMed Central PMCID: PMC4348198. 25734259 PMC4348198

[34] Vera-Arias CA, Holzschuh A, Oduma CO, Badu K, Abdul-Hakim M, Yukich J, et al. High-throughput Plasmodium falciparum hrp2 and hrp3 gene deletion typing by digital PCR to monitor malaria rapid diagnostic test efficacy. Elife. 2022;11. Epub 2022/06/29. doi: 10.7554/eLife.72083 . 35762586 PMC9246365

[35] Cheng Q, Gatton ML, Barnwell J, Chiodini P, McCarthy J, Bell D, et al. Plasmodium falciparum parasites lacking histidine-rich protein 2 and 3: a review and recommendations for accurate reporting. Malar J. 2014;13:283. Epub 2014/07/24. doi: 10.1186/1475-2875-13-283 ; PubMed Central PMCID: PMC4115471. 25052298 PMC4115471

[36] Simundic AM. Measures of Diagnostic Accuracy: Basic Definitions. EJIFCC. 2009;19(4):203–11. Epub 2009/01/01. ; PubMed Central PMCID: PMC4975285. 27683318 PMC4975285

[37] United Nations Department of Economic and Social Affairs PD. World Population Prospects 2019, Volume II: Demographic Profiles: Burundi 2019. Available from: https://population.un.org/wpp/Graphs/1_Demographic%20Profiles/Burundi.pdf.

[38] Koepfli C, Nguitragool W, Hofmann NE, Robinson LJ, Ome-Kaius M, Sattabongkot J, et al. Sensitive and accurate quantification of human malaria parasites using droplet digital PCR (ddPCR). Sci Rep. 2016;6:39183. Epub 2016/12/17. doi: 10.1038/srep39183 ; PubMed Central PMCID: PMC5159915. 27982132 PMC5159915

[39] Jang IK, Tyler A, Lyman C, Kahn M, Kalnoky M, Rek JC, et al. Simultaneous Quantification of Plasmodium Antigens and Host Factor C-Reactive Protein in Asymptomatic Individuals with Confirmed Malaria by Use of a Novel Multiplex Immunoassay. J Clin Microbiol. 2019;57(1). Epub 2018/11/09. doi: 10.1128/JCM.00948-18 ; PubMed Central PMCID: PMC6322473. 30404944 PMC6322473

[40] Hosch S, Yoboue CA, Donfack OT, Guirou EA, Dangy JP, Mpina M, et al. Analysis of nucleic acids extracted from rapid diagnostic tests reveals a significant proportion of false positive test results associated with recent malaria treatment. Malar J. 2022;21(1):23. Epub 2022/01/26. doi: 10.1186/s12936-022-04043-7 ; PubMed Central PMCID: PMC8785039. 35073934 PMC8785039

[41] Meatherall B, Preston K, Pillai DR. False positive malaria rapid diagnostic test in returning traveler with typhoid fever. BMC Infect Dis. 2014;14:377. Epub 2014/07/10. doi: 10.1186/1471-2334-14-377 ; PubMed Central PMCID: PMC4094604. 25005493 PMC4094604

[42] Gatton ML, Ciketic S, Barnwell JW, Cheng Q, Chiodini PL, Incardona S, et al. An assessment of false positive rates for malaria rapid diagnostic tests caused by non-Plasmodium infectious agents and immunological factors. PLoS One. 2018;13(5):e0197395. Epub 2018/05/15. doi: 10.1371/journal.pone.0197395 ; PubMed Central PMCID: PMC5951549. 29758050 PMC5951549

[43] Lee JH, Jang JW, Cho CH, Kim JY, Han ET, Yun SG, et al. False-positive results for rapid diagnostic tests for malaria in patients with rheumatoid factor. J Clin Microbiol. 2014;52(10):3784–7. Epub 2014/07/25. doi: 10.1128/JCM.01797-14 ; PubMed Central PMCID: PMC4187788. 25056333 PMC4187788

[44] Bousema T, Okell L, Felger I, Drakeley C. Asymptomatic malaria infections: detectability, transmissibility and public health relevance. Nat Rev Microbiol. 2014;12(12):833–40. doi: 10.1038/nrmicro3364 . 25329408

[45] Koepfli C, Ome-Kaius M, Jally S, Malau E, Maripal S, Ginny J, et al. Sustained Malaria Control Over an 8-Year Period in Papua New Guinea: The Challenge of Low-Density Asymptomatic Plasmodium Infections. J Infect Dis. 2017;216(11):1434–43. Epub 2017/10/14. doi: 10.1093/infdis/jix507 ; PubMed Central PMCID: PMC5853328. 29029179 PMC5853328

