# Supplementary material for: Performance of highly sensitive and conventional rapid diagnostic tests for clinical and subclinical Plasmodium falciparum infections, and hrp2/3 deletion status in Burundi
Source: PLOS Glob Public Health. 2022 Jul 28;2(7):e0000828. doi: 10.1371/journal.pgph.0000828 (PMC10022336; doi:10.1371/journal.pgph.0000828)
Supplement: S1 File — (DOCX) [file pgph.0000828.s001.docx]

**Performance of highly sensitive and conventional rapid diagnostic tests for clinical and subclinical *Plasmodium falciparum* infections, and *hrp2*/*3* deletions status in Burundi**

David Niyukuri, Denis Sinzinkayo, Emma V. Troth, Colins O. Oduma, Mediatrice Barengayabo, Mireille Ndereyimana, Aurel Holzschuh, Claudia A. Vera-Arias, Yilekal Gebre, Kingsley Badu, Joseph Nyandwi, Dismas Baza, Elizabeth Juma, Cristian Koepfli

**Supplementary File S1: Laboratory Protocols**

**A) *Plasmodium falciparum* *var*ATS qPCR**

Reference: Ultra-sensitive detection of *Plasmodium falciparum* by amplification of multi-copy subtelomeric targets.

Hofmann N, Mwingira F, Shekalaghe S, Robinson LJ, Mueller I, Felger I

PLoS Medicine 2015

All primers and probes a 10 µM

qPCR master mix used: QuantaBio PerfeCTa qPCR ToughMix (catalogue no. 95112-012). Alternatively, the ThermoFisher TaqMan FastAdvanced Master Mix (catalogue no. 4444556) can be used.

Reaction mix setup

varATS forward+reverse primers 0.48 µL

varATS_probe 0.48 µL

PerfeCTa Though Mix 6 µL

H_2_O 1.04 µL

DNA 4 µL

Total 12 µL

Cycling conditions

50° 2 min

95° 2 min

95° 10 sec

55° 30 sec 45 cycles

Primer and probe sequences

Pf_varATS forward CCCATACACAACCAAYTGGA

Pf_varATS reverse TTCGCACATATCTCTATGTCTATCT

Pf_varATS probe 6-FAM-TRTTCCATAAATGGT-NFQ-MGB

**B) *hrp2* exon 2/tRNA ddPCR protocol**

Reference: High-throughput *Plasmodium falciparum hrp2* and *hrp3* deletion typing by digital PCR to monitor malaria rapid diagnostic test efficacy

Claudia A. Vera-Arias, Aurel Holzschuh, Colins O. Oduma, Kingsley Badu, Mutala Abdul-Hakim, Joshua Yukich, Manuel W. Hetzel, Bakar S. Fakih, Abdullah Ali, Marcelo U. Ferreira, Simone Ladeia-Andrade, Fabián E. Sáenz, Yaw Afrane, Endalew Zemene, Delenasaw Yewhalaw, James W. Kazura, Guiyun Yan, Cristian Koepfli

eLife 2022

Instrumentation

BioRad QX200 ddPCR system

Reaction mix setup

All primers and probes a 10 µM

SuperMix for Probes (no dUTP) 11 µL Supplier: BioRad

hrp2_fwd 1.76 µL

hrp2_rev 1.76 µL

hrp2_Probe 0.88 µL

tRNA_fwd 0.44 µL

tRNA_rev 0.44 µL

tRNA_Probe 0.22 µL

H_2_O 3.5 µL

DNA 2 µL

Cycling conditions

95° 10 min

94° 30 sec

56° 1 min 45 cycles

98° 10 min

Primer and probe sequences

hrp2_forward CATTTTTAAATGCTTTTTTATTTTTATATAG

hrp2_probe FAM-CGCATTTAATAATAACTTGTGTAGCAAAAATGC-BHQ1

hrp2_reverse CTTGAGTTTCGTGTAATAATCTC

tRNA_Probe HEX-CTA CCT CAG AAC AAC CAT TAT GTG CT-BHQ1

tRNA_fwd CAT CAA ATG AAG ATT TAA CAA GAG

tRNA_rev CTT TTT GAT TCT ATA GTT TCA TCT TTA TG

**C) *hrp3*/*tRNA* ddPCR protocol**

Reference: High-throughput *Plasmodium falciparum hrp2* and *hrp3* deletion typing by digital PCR to monitor malaria rapid diagnostic test efficacy

Claudia A. Vera-Arias, Aurel Holzschuh, Colins O. Oduma, Kingsley Badu, Mutala Abdul-Hakim, Joshua Yukich, Manuel W. Hetzel, Bakar S. Fakih, Abdullah Ali, Marcelo U. Ferreira, Simone Ladeia-Andrade, Fabián E. Sáenz, Yaw Afrane, Endalew Zemene, Delenasaw Yewhalaw, James W. Kazura, Guiyun Yan, Cristian Koepfli

eLife 2022

Instrumentation

BioRad QX200 ddPCR system

Reaction mix setup

All primers and probes a 10 µM

SuperMix for Probes (no dUTP) 11 Supplier: BioRad

hrp3_fwd 0.66

hrp3_rev 0.66

hrp3_Probe 0.44

tRNA_fwd 0.44

tRNA_rev 0.44

tRNA_Probe 0.22

H_2_O 6.14

DNA 2

Cycling conditions

95° 10 min

94° 30 sec

56° 1 min 45 cycles

98° 10 min

Primer and probe sequences

Hrp3_fwd ATGCTAATCACGGATTTCATTTTA

Hrp3_Probe            FAM-CCTTCACGATAACAATTCCCATACTTTAC

Hrp3_rev                 ATCGTCATGGTGAGAATCATC

tRNA_Probe HEX-CTA CCT CAG AAC AAC CAT TAT GTG CT-BHQ1

tRNA_fwd CAT CAA ATG AAG ATT TAA CAA GAG

tRNA_rev CTT TTT GAT TCT ATA GTT TCA TCT TTA TG
