# Supplementary material for: Performance of highly sensitive and conventional rapid diagnostic tests for clinical and subclinical Plasmodium falciparum infections, and hrp2/3 deletion status in Burundi
Source: PLOS Glob Public Health. 2022 Jul 28;2(7):e0000828. doi: 10.1371/journal.pgph.0000828 (PMC10022336; doi:10.1371/journal.pgph.0000828)
Supplement: Attachment — Submitted filename: Burundi_RDT_Response_To_reviewers.docx [file pgph.0000828.s003.docx]

We thank the reviewers for their detailed comments and suggestions for improvement. We are confident we were able to address all comments.

Reviewer comments are in black.

Our responses are in blue.

Question 3: Have the authors made all data underlying the findings in their manuscript fully available (please refer to the Data Availability Statement at the start of the manuscript PDF file)?

Reviewer #1: No

Reviewer #2: Yes

Reviewer #3: Yes

Answer: All data that was used for the analysis presented in the manuscript (age, sex, RDT and qPCR positivity) is provided in supplementary File S1. We do not know what other data reviewer 1 would like to see.

Reviewer #1: Title: A novel rapid diagnostic test for highly sensitive diagnosis of clinical and subclinical Plasmodium falciparum infections
Authors: Niyukuri et al.

Comments
Abstract: Although the results (sensitivity) of the novel Rapigen Biocredit three-band Plasmodium falciparum HRP2/LDH RDT for clinical (febrile) and subclinical individuals were compared against AccessBio CareStart HRP2 RDT, the result doesn’t include the performance of novel Rapigen Biocredit three-band Plasmodium falciparum HRP2/LDH RDT performance against qPCR in the abstract section.

Answer: All sensitivities in the abstract are compared to qPCR as gold standard. We have clarified this as follows (lines 30-31):

“Sensitivity compared to qPCR among clinical patients was […]
The terms clinical and febrile seem interchangeably used. It would be nice if authors consistently use either in the text.

Answer: We have changed the term to ‘clinical’ wherever appropriate.
There is no mention on the recruitment processes of 444 clinical and 468 subclinical individuals. What specific tools were used to diagnose malaria at those health facilities? Were treatments given to those tested positive for malaria?

Answer: We do not believe that these details should be part of the abstract. We have added the following clarification to the methods section (lines 123-124):

“Individuals found positive were treated according to national guidelines.”

Line 14, ………… present challenges for RDT-based diagnosis. This should read as ‘….present challenges to HRP2/3-based RDTs.
Answer: This sentence reads: Low-density infections, and deletions of the *P. falciparum hrp2*/*3* genes (encoding the HRP2 and HRP3 proteins detected by many RDTs) present challenges for RDT-based diagnosis.”

Low-density infections present challenges to any RDT-based diagnosis, thus the sentence is correct as stated.

Introduction
It is well written.

MM
Rapid diagnostic tests: the description presented under this subheading shall be written under the introduction section. This section perhaps should describe how the authors have conducted the evaluation instead of narrating the use of different RDTs and the significance of the different target antigens to the parasite. The lot number and other important parameters of the RDTs are not well stated.

Answer: We feel this section is too specific to be included in the introduction, and better in placed in the methods section.

We have added detail on how we evaluated the performance characteristics, please see our detailed answer below.

We have added the lot numbers for both tests.

Authors shall report the situation of malaria (including species composition) in the study area so that endemicity of the area can be better understood. Indeed, the fact that market places were used for the recruitment of subclinical individuals, there was no justification for choosing such places although there is a mention on using convenience sampling strategy.

Answer: To the best of our knowledge this is the first epidemiological study in this site, and no data on species composition is available. We plan to screen samples for other *Plasmodium* species eventually, these results will be reported separately.

As explained in the methods section, we used a convenience sampling strategy. Considering logistics and safety of the study team, sampling on the market was considered the best option for this study. Unfortunately, given the situation in the site, the study team did not feel safe visiting individual households. The samples consisted of a fair representation of the population from various sub villages of the community.

Sample collection and RDT diagnosis
The clinical samples were collected in self-reported febrile patients at health-facilities; however, the subclinical individuals were recruited from market. Why the market place is chosen?

Answer: As explained above, this was the most feasible option.

Line 108/109: …….’Only 27/468 subclinical individuals were children below 5 years of age, and only 73/468 were children aged 5- 109 15 years’. The majority of those sampled were female. These sentences are wrongly placed under this section (MM) as they describe results and should be moved to the result section. Moreover, Table 1 also describes results and hence shall move to the appropriate place, result. Caption should be written above the table.
This section is haphazardly written and shall be revised accordingly.

Answer: We moved these sentences to the results section (lines 206-210). Note that the final typesetting of the manuscript will be done by the journal and we cannot determine where table 1 will be placed.

Line 128: P. falciparum qPCR and hrp2/3 deletion typing
Authors shall describe how they collected blood samples before embarking on the procedures used for DNA extraction, qPCR and hrp2/3 gene del. What is the expected bp of hrp2 exon2?

Answer: The assay used is described in detail in our previous publication that we have referenced (reference 30). We have included the protocol in supplementary File S1 and added the following detail to the manuscript (lines 166-171):

“In this assay, *hrp2* or *hrp3* and a control gene (*serine-tRNA ligase*) are quantified in a single tube with very high specificity, thus providing highly accurate data on deletion status [30]. The assay amplifies parts of *hrp2* exon 2 that encodes for the antigen detected by the RDT. The amplified region is deleted in all reported cases where *hrp2* deletion breakpoints were studied [31]. Assay conditions are given in supplementary File S1.”

Did authors use microscopy for the detection and quantification of parasitaemia?. Details are needed.

Answer: No microscopy was done as part of this study.

Details of the primers used for qPCR and the cycling conditions are not described.

Answer: We have added these details as supplementary File S1 and added a note to lines 159:

“qPCR conditions are given in supplementary File S1.”

Details on how authors did hrp2/3 gene deletion are needed. Did authors amplify exon 2, 3 including the upstream and downstream flanking regions? Assessment of hrp2/3 deletion should encompass the entire coding region. ddPCR is inadequately presented.

Answer: As explained above, this data has been added to the supplementary file.

How the sample size (444 clinical and 468 subclinical) was calculated?

Answer: We have added our sample size calculation (lines 174-177):

“The sample size was determined to be able to detect a 5% difference in sensitivity with 95% confidence. In the absence of any preliminary data, a prevalence of infection of 50% was assumed, yielding the largest sample size of n=377. The number of individuals sampled (n=444 clinical and n=468 subclinical) was above the minimum target, these numbers were reached out of convenience.”

Results
In Table 1 (line 117-118), the test positivity by qPCR, novel RDT, the conventional RDT (CareStart RDT) shall be reported in such a way that the results could be easily accessed.

Answer: Test positivity by qPCR and both RDTs is presented in Table 2. We feel Table 1 will become difficult to read If further data are included. As the main focus of this manuscript is the evaluation of the Rapigen Biocredit RDT, we feel it is appropriate to only include these data.

It is unclear why authors did not use microscopy as part of the comparison of the different malaria diagnostic tools/

Answer: Unfortunately, expert microscopy was not available for this study. This study was conducted at a time where covid travel restrictions prevented any US-based investigator from traveling to the field site, and it was not possible to train local researchers remotely in how to prepare slides for expert microscopy, and have them shipped to a lab with certified microscopists.

Table 2 (155-156): the LoD (parasites/uL) for the different types of RDTs presented in the table is not well reviewed in the background information. References are needed as far as LoD is concerned for those RDTs. The caption of table two needs revision to conform to the message it conveys (clinical and subclinical samples).

Answer: For clarification, these data is from our study, thus there are no references that can be added.
The calculation of sensitivity and specificity of the RDTs should be compared against the qPCR, so that the activity of each of the RDT can be appreciated.

Answer: We have clarified the caption as follows:

“Limit of detection, AUC, and sensitivity and specificity of RDTs as compared to qPCR”

Line 164-165: it reads ‘the sensitivity of the Biocredit test was 72.3% (162/238, Figure 165 1A, Table 2)’, however, this value is incorrect since 162/238 gives 68.1%, but not 72.3%. Instead, the sensitivity of the Biocredit could be 162/224.

Answer: Thank you for spotting this error, the reviewer is correct. The correct sentence now reads:

“The sensitivity of the Biocredit test was 72.3% (162/224, Figure 1A, Table 2).”

The sensitivity and specificity of the Biocredit can be calculated using 2 x 2 tables, so that the results of the Biocredit against qPCR can easily be appreciated.

Answer: We have added 2x2 tables (Table 3)

Line 168-169: I am not sure whether authors used novel Biocredit RDT or CareStart RDT as a reference while calculating sensitivity. The sensitivity of the Biocredit RDT and CareStart RDT shall be compared against the reference test (qPCR).

Answer: We compared each RDT to qPCR. We have added this to line 211:

“[…] compared to qPCR”

The results displayed in Fig.2 are not readable and needs revision.

Answer: We have increased the font size.

Discussion
It is interesting to note that qPCR detected falciparum malaria in 70.5% (313/444) of the clinical samples and 47.9% (224/468) malaria in the subclinical samples. Is there any data on the prevalence of subclinical malaria in the study area before?

Answer: To the best of our knowledge, this is the first study on subclinical malaria in this site.

In line 218-219, authors report that the novel RDT detected 80% of clinical and 72% of the subclinical infections. What about CareStart RDT? The prevalence of clinical and subclinical is comparable and this needs strong justifications to be valid.

Answer: Detailed data for the CareStart RDT are given in the results section and Table 2. The focus of this study is the evaluation of the Rapigen RDT. We thus do not believe it is warranted to repeat the CareStart results here.

It is not unexpected that in high transmission settings a large proportion of subclinical infections can be detected by microscopy or RDT (see e.g. Figure 1 in <https://www.nature.com/articles/nrmicro3364> (reference 42 in our manuscript).

Authors did not discuss the likely reasons for detecting 82.8% of the clinical and 63% of subclinical samples were tested qPCR positive among 5-15 years.

Answer: We have added the following detail in the discussion (line 302-303):

“Due to the acquisition of natural immunity after repeated infections, parasite densities in children are highest, they are at highest risk of severe malaria, and in high transmission settings they are an important reservoir of transmission.”

Reviewer #2: Review of the manuscript: “A novel rapid diagnostic test for highly sensitive diagnosis of clinical and subclinical Plasmodium falciparum infections” (PGPH-D-22-00743)


Summary: This article assessed the performance of a novel P. falciparum specific lateral flow assay against a commercially available rapid diagnostic test and qPCR with a theoretical LOD of <0.1 parasites / µl. Through non-random selection procedures a total of 444 clinical Pf patients and 468 asymptomatic participants were enrolled. Sensitivity of the novel diagnostic against qPCR was 80% and 73% for clinical and asymptomatic participants respectively. The novel diagnostic showed better performance than the commercially available RDT>

Major Comment: This is a well written article covering a topic of public health. My main concern is that the authors did not calculate the area under the ROC curve, which would have made it much easier to compare performance of both RDTs directly as well as both antigens.

Answer: We have included AUC for all RDTs in Table 2.

In addition, the statistical methods as stated right now are not sufficient. In case the authors do not want to compare the areas under the ROC, they at least need to clarify in the methods what approach they have used to compare performance?

Answer: We have rewritten the paragraph on performance characteristics analyzed (lines 178-194):

“The following performance characteristics were determined for both RDTs, and the HRP2 and LDH bands on the Biocredit RDT separately as well as in combination: (i) sensitivity: the number of infections detected by an RDT divided by the total number of infections detected by qPCR as gold standard. (ii) Specificity: the proportion of negative RDTs among individuals that tested negative by qPCR. (iii) Limit of detection (LoD): LoD was defined as the lowest parasite density where a qPCR-positive infection would be detected with 95% probability. To determine the limit of detection (LoD) of each RDT target, logistic regression analysis was conducted. (iv) Area under the receiver operating characteristic curve (ROC) curve (AUC): AUC was calculated with a nonparametric analysis using 1000 bootstrap replications, with log10 transformed parasite density by qPCR as classification variable. Diagnostic accuracy was considered excellent if AUC was >0.9, and very good for AUC values of >0.8 to <0.9 [34].“

Finally the authors draw conclusions about the prevalence of sub-clinical malaria within the local population (in lines 256 and thereafter), considering the sampling method such a conclusion cannot be made.

Answer: we have clarified that our statement refers to the study population and might not be representative for the entire country (lines 342-343):

“Half of the mostly adult study population in the study site in Burundi’s north-west carried subclinical *P. falciparum* infections.”

Other comments:
• Can you provide some background on the novel RDT in the introduction? For example:
o Test procedures
o Time to result
o Costs

Answer: We have added the following detail to the methods section (lines 113-116):

“Testing procedures are identical for both RDTs and are explained in detail e.g. in [31]. A droplet of blood (approximately 5 µL), collected either by finger prick or phlebotomy, is put onto a specific spot on the test kit, followed by 2-3 drops of assay diluent buffer on a separate spot. Results are read after 15-20 minutes. The cost for either test is approximately 1 USD.”

• Table 1 contains a large proportion of all results, however is located within the methods section, consider moving this table to the results section?

Answer: We moved the section on the demographic composition to the results, thus table 1 is now mentioned for the first time in the results section. Please note that final typesetting will be done by the journal and we do not have any power on where tables will be located.

Minor Comments:
• Kindly include assay specific specificity in the abstract as well

Answer: We added specificity data to the abstract for clinical and subclinical individuals:

Lines 32-33:

“Specificity of the Biocredit was 82.4% compared to 96.2% for CareStart.”

Lines 34-35:

“Specificity was 84.4% for the Biocredit and 93.4% for the CareStart RDT.”

• In line 42 the authors suggest that sensitivity of RDTs is superior to light microscopy and it would be good if this statement was backed up by a reference.

Answer: We have added two references. We have also clarified that we refer to field microscopy, which tends to be less sensitive that expert microscopy (line 47).

• Line 68: this sentence needs to be revised for grammar

Answer: we have re-phrased this sentence as follows (line 75):

“The level of malaria transmission in Burundi is among the highest in the world.”

• Lines 72 – 75: this section appears more suitable with the methods section?

Answer: We disagree with this comment. It is common practice that the last paragraph of the introduction very briefly prepares the reader for what he/she will be presented with.

These lines read (lines 79-82):

“To understand the benefit a novel, highly sensitive RDT, suspected febrile cases presenting to a health center, and subclinical community members were tested by the novel RDT, an established RDT, and results compared to highly sensitive qPCR. *P. falciparum* positive infections were typed for *hrp2* and *hrp3* deletions.”

We believe the end of the introduction is the correct place for these lines.

• Line 97: can you clarify if the Carestart RDT is WHO pre-qualified – I think it is?

Answer: We have clarified that the test is pre-qualified (lines 109-110):

“This HRP2-based RDT is WHO pre-qualified [26]”

• Lines 107 – 109 are results and seem better placed in the results than methods section

Answer: We moved these lines to the results.

• Line 126: How long did it take on average before samples were stored at -20°C?

Answer: We have clarified this as follows (line 150)

“Remaining blood was placed into -20°C storage every evening and kept at -20°C until DNA extraction.”

• Line 144: there is a funny sentence around CI, which should probably not be there?

Answer: Yes, this sentence should be there. It explains that what the acronym CI95 stands for. There are different acronyms for the confidence interval used in the literature, thus it is important to define it. We have changed it to (line 197):

“CI95 stands for 95% confidence interval.”

• Kindly extend the methods and describe briefly how performance was calculated

Answer: We have added the following paragraph (line 178-194):

“The following performance characteristics were determined for both RDTs, and the HRP2 and LDH bands on the Biocredit RDT separately as well as in combination: (i) sensitivity: the number of infections detected by an RDT divided by the total number of infections detected by qPCR as gold standard. (ii) Specificity: the proportion of negative RDTs among individuals that tested negative by qPCR. (iii) Limit of detection (LoD): LoD was defined as the lowest parasite density where a qPCR-positive infection would be detected with 95% probability. To determine the limit of detection (LoD) of each RDT target, logistic regression analysis was conducted. (iv) Area under the receiver operating characteristic curve (ROC) curve (AUC): AUC was calculated with a nonparametric analysis using 1000 bootstrap replications, with log10 transformed parasite density by qPCR as classification variable. Diagnostic accuracy was considered excellent if AUC was >0.9, and very good for AUC values of >0.8 to <0.9 [34].“

• Table 2: add 95% confidence intervals to performance indicators

Answer: We have added confidence intervals to all values.

• Lines 219 – 220: please use the full name of either diagnostic rather than talking about an “established test”

Answer: We have added the names to this sentence (lines 299-300):

“The novel Biocredit RDT performed substantially better compared to the established CareStart RDT that is used by malaria control programs in multiple countries.”

• Lines 244 – 245 states that countries switching from HRP2 based RDTs to non-HRP2 diagnostics can expect a comparable performance. However, from the presented data it appears that sensitivity dropped from 77% to 72%, which seems like quite a lot. Kindly use a sloid statistical method to determine whether this difference is significant and discuss accordingly

Answer: Our statement refers to countries currently using the Carestart HRP2 RDT (not the Rapigen HRP2 RDT with a sensitivity of 77%). The sensitivity among qPCR-positive clinical individuals was 71.88% (225/313) for the Rapigen LDH band, and 73.16% (229/213) for Carestart. We have clarified this sentence as follows (lines 330-332):

“Countries currently using the Carestart HRP2 RDT that switch to non-HRP2-based RDTs due to high levels of deletion can expect similar sensitivity of diagnosis in clinical settings using the new test as with previous HRP2-based RDTs.”

• Line 250: clarify that by antigen you mean HRP2

Answer: Any antigen can persist after treatment, not only HRP2. Thus, the sentence as written is correct.

• Line 256 states that half the population carried sub-clinical Pf infections. Given the non-random nature of sampling this statement cannot be made, kindly remove.

Answer: we have clarified that our statement refers to the study population and might not be representative for the entire country (line 342):

“Half of the mostly adult study population in the study site in Burundi’s north-west carried subclinical *P. falciparum* infections.”

Reviewer #3: The manuscript presented by the authors contains interesting and important information for malaria research community and also for the malaria control programs that are looking for new/better diagnostic tools. However there are some comments to the authors and some corrections that need to be addressed before the publication:

1. The title mention "A novel rapid diagnostic test..."; but looking at the literature there is a paper from 2020 where the authors compare 3 commercial RDTs from BIOCREDIT, including the Pf (HRP2/LDH). This paper, by Seo Hye Park et al. (Korean J Parasitol. 2020 Apr; 58(2): 147–152. Published online 2020 Apr 30. doi: 10.3347/kjp.2020.58.2.147), should be included in the discussion also. In addition, these tests were also part of the Malaria Rapid Diagnostic Test Performance-Results of WHO product testing of malaria RDTs: round 6 (2014-2015).
Could the authors please confirm if the test they used are the same (just different lot) or is it a new (improved) version?, if so, please give more details about it and also include lot number.

Answer: We have added this reference and brief description of the study (lines 102-105):

“The test was evaluated previously among microscopy-positive individuals and showed very high agreement with diagnosis by microscopy [23]. It has not been not evaluated among clinical patients that might harbor submicroscopic infections, or subclinical individuals.”

Please note that in this previous study only microscopy-positive and true negative individuals were tested (the abstract mentions nested PCR, but Table 2 shows that all individuals positive by nested PCR were also positive by microscopy). Thus, this study does not represent a scenario where many infections are below the limit of detection of microscopy.

2. In the summary (line 31) it is mention ..."and reached 88.3% (53/60) in children < 15 years". By other hand in the results section (line 207) it says..."sensitivity was 88.3% (53/61) in children"...
Please correct the number: 60 or 61 children < 15 years?.

Answer: Thank you for spotting this typo. The correct number is 53/60, we have corrected it.

3. Table 1 is named "Demographics of study population and risk factors for infection (by qPCR and RDT)"...The percentage of each age group should be included and also the percentage of male and female. In addition, it is not explain how do the did the analysis of risk factors. Finally, if the authors want to keep "test positivity and prevalence by qPCR and RDT", I suggest to include also the data on specificity.

Answer: We have included percentages.

We have changed the caption of the Table 1 to (line 127-128):

**“Table 1**: Demographics of study population and prevalence and test positivity (by qPCR and RDT) by age group and gender.”

We have also clarified in the table legend (lines 146-147):

“*P*-values to compare groups were calculated by Chi-square test.”

Specificity is presented in Table 2. We feel Table 1 will be difficult to read if it is included there as well.

4. This paper also includes data on hrp2/3 deletion typing, an important issue in African endemic countries, so this should be mention in the objectives and, if possible, also in the title.

Answer: We have changed the title as follows (lines 1-2):

“Performance of highly sensitive and conventional rapid diagnostic tests for clinical and subclinical *Plasmodium falciparum* infections, and *hrp2*/*3* deletion status in Burundi”

5. In the conclusion, in addition to mention the high sensitivity for the diagnosis of clinical and subclinical infections, it should also mention the absent of hrp2 deletion. The rest of the text in this paragraph (lines 265-267) could be merged with the previous paragraph (lines 256-262) as part of the discussion and future studies.

Answer: We have moved the following statement to the conclusions (lines 355-357)

“Given the absence of *hrp2* deletions, HRP2-based diagnostic remains appropriate in the site of the current study. Surveillance for hrp2 and hrp3 deletions will be required in other sites across Burundi.”

There seems to be a confusion, as lines 265-267 (now lines 342-246) are not part of the conclusions.
